# Development, validation, and reliability of the Chrononutrition Profile Questionnaire-Pregnancy (CPQ-P)

**DOI:** 10.1186/s12884-024-06403-4

**Published:** 2024-03-23

**Authors:** Ee Yin Kok, Satvinder Kaur, Nurul Husna Mohd Shukri, Nurliyana Abdul Razak, Masaki Takahashi

**Affiliations:** 1 Department of Food Science and Nutrition, Faculty of Applied Sciences, UCSI University, Cheras, Wilayah Persekutuan Kuala Lumpur, 56000 Malaysia; 2 Department of Nutrition, Faculty of Medicine and Health Sciences, University Putra Malaysia, Selangor, Malaysia; 3 Institute for Liberal Arts, Tokyo Institute of Technology, Tokyo, Japan

**Keywords:** Chrononutrition, Maternal health, Circadian rhythm, Eating misalignment, Sleep

## Abstract

**Background:**

During pregnancy, physiological changes can affect eating and sleeping habits, which may eventually have negative consequences for maternal and foetal health. To better understand these changes, it is essential to develop a reliable questionnaire that addresses lifestyle habits such as snacking and daytime napping. This study aimed to determine the validity and reliability of the Chrononutrition Profile Questionnaire-Pregnancy (CPQ-P).

**Methods:**

A total of 399 women in their second and third trimester of pregnancy were recruited from government maternal and child health clinics in Kuala Lumpur and Putrajaya and completed a self-administered online questionnaire. Content validity was conducted with an expert panel consisting of 4 members. Confirmatory factor analysis (CFA) using maximum likelihood was conducted to determine the construct validity. Internal consistency was determined by Cronbach’s alpha coefficient (CAC), while the test-retest reliability was conducted using intraclass correlation coefficient (ICC).

**Results:**

The questionnaire had an appropriate content validity index of 0.91. The CPQ-P consists of 22 items, measuring 5 constructs, including morning chrono-habits, sleeping habits, evening eating, temporal eating, and pregnancy symptoms. The factor model showed good fit with χ^2^/df = 2.486, GFI = 0.893, CFI = 0.912, and RMSEA = 0.065. The 22 items in CPQ-P showed fair to excellent test-retest reliability (ICC: 0.42 to 0.98). The 5 constructs in CPQ-P were found to have a good to excellent internal consistency (α = 0.612–0.963).

**Conclusions:**

The CPQ-P is a valid and reliable tool for assessing lifestyle habits during pregnancy. The questionnaire can be used to identify areas where pregnant women may need additional support or intervention to adopt healthy behaviours and reduce the risk of adverse maternal and foetal outcomes.

**Trial registration:**

NCT05700136 (clinicaltrials.gov). Trial registration date: 26/01/2023.

**Supplementary Information:**

The online version contains supplementary material available at 10.1186/s12884-024-06403-4.

## Introduction

In recent years, chrononutrition has been studied to investigate the impact of meal timing and circadian rhythms on health. It has been shown that the timing of food intake is equally important as the quantity and amount of food consumed because our circadian rhythms dictate the metabolism of nutrients in our body [1]. Our circadian clock in the hypothalamic suprachiasmatic nuclei (SCN) is entrained by external cues such as the 24-hour light dark cycle and timing of food intake [2]. When there is a disruption or misalignment of our circadian rhythm, it may cause adverse effects on our metabolic health. Metabolic rhythms such as glucose homeostasis, insulin regulation, and energy expenditure are in favour of earlier meal timings, characterized by larger distribution of energy intake on the earlier window of the day [2].

Pregnancy is a crucial period of development for both the mother and the growing fetus. Adequate nutrition plays a vital role in supporting maternal health, promoting optimal fetal growth, and reducing the risk of complications such as preterm birth, low birth weight, and small for gestational age [3]. The Barker’s hypothesis states that a malnutrition state during pregnancy would lead to unfavourable health outcomes of the offspring during adulthood such as obesity and cardiovascular diseases [4]. Hence, the nutritional status of the mother during pregnancy can have long-lasting effects on the health and well-being of both mother and child.

Chrononutrition practices such as breakfast skipping, long eating window, and night eating have been found to influence both the mother and the infant’s outcomes [5, 6]. As diet is one of the factors synchronizing our circadian rhythm, irregular meal timings can be disruptive to the alignment of environmental oscillators with the central pacemaker, resulting in adverse health outcomes [7]. For example, breakfast skipping among pregnant women in Japan has been associated with low infant birth weight compared to those who eat breakfast daily [8]. Eating window and breakfast skipping was also associated with melatonin and cortisol rhythm, which ultimately increases the risk of pre-eclampsia and intrauterine growth retardation [9]. A study done in Singapore had reported that pregnant women had a high prevalence of meal skipping and meal delaying (28% and 29% respectively), and it has been correlated with poor sleep and emotion [10]. Notably, breakfast skipping was examined in most chrononutrition-related studies, as it is the first meal taken in a day [11, 12]. Gontijo et al. (2018) reported that an earlier breakfast, longer eating window, and more meals taken in a day was associated with improved diet quality during pregnancy, which can be favourable for the mother’s health [13].

Pregnant women also experience changes in sleep patterns and disorders such as insomnia, nocturnal awakening, and restless legs syndrome due to hormonal changes [14]. These factors causing sleep disturbance may negatively affect the sleep quality of pregnant women and leads to adverse outcomes such as preterm birth, maternal psychological distress, and increased risk of childhood obesity [15, 16]. Sleep disturbances during pregnancy has also affected the sleep and wake time, which subsequently affects their meal timings throughout the day [17]. In order to overcome the sleep debt from disturbed night time sleep, many pregnant women take naps during the day and it was found that napping for approximately 1.5 h during pregnancy could contribute to reduce risk of low birth weight infants [18]. Hence, pregnancy symptoms should be considered to fully represent the pregnancy condition which affects lifestyle habits.

Despite the growing interest in the field of chrononutrition, there is a lack of standardized questionnaires specifically designed to capture meal timing data and its relationship with pregnancy-related variables. Most studies use a diet record and sleep questionnaire to capture meal and sleep timings during pregnancy [9, 19]. This may reduce the compliance rate as the questionnaires are lengthy and may require trained interviewers to capture the accurate data. To investigate the association between meal timing and pregnancy outcomes, it is essential to have a reliable and validated tool to assess chrononutrition patterns in pregnant women. Developing and validating such a questionnaire would enable researchers and healthcare providers to comprehensively assess chrononutrition patterns in pregnant women. To the best of our knowledge, this was the first validated questionnaire to determine the chrononutrition profile of pregnant women. The current CPQ-P addresses additional items related to pregnancy symptoms, snacking habits, and daytime napping which may represent changes during the gestation period. Hence, this study aims to modify, validate, and test the reliability of the Chrononutrition Profile Questionnaire-Pregnancy (CPQ-P). This is part of a longitudinal study (clinicaltrials.gov: NCT05700136, registered on 26/01/2023) which objective is to determine the prenatal and postnatal factors associated with infant circadian rhythm, growth, and temperament.

## Methodology

### Study design and population

A total of 399 participants were recruited from government maternity clinics in Kuala Lumpur and Putrajaya, Malaysia. The inclusion criteria were Malaysian pregnant women aged 18–39 years old, literate in English or Bahasa Malaysia, not having any health comorbidities before or during pregnancy, and not under any medications related to sleep. Participants were excluded if they were working night shift or diagnosed with any sleep disorders. The recruitment commenced from 6th September 2022 to 17th November 2022. The study protocol was reviewed and approved by the Medical Research Ethics Committee (NMRR ID-22-00182-HIP (IIR)).

### Sample size calculation

For overall testing and analysis, at least 10 participants were required for each item included in the questionnaire [20]. Hence, a total of 290 participants was required for the validation of the current questionnaire.

$$N=10 \times 29\,items=290\,participants$$

To compensate for 20% non-compliance and non-response rate, the total sample size required was 348 participants.

To perform test-retest analysis, with an effect size of 0.8, alpha level of 0.05, the minimum sample size required was 22 subjects to obtain ICC value of 0.5. After accounting for 20% dropout rate, a total of 28 subjects should be recruited [21].

### Study procedures

Participants were recruited from nine government Maternal and Child Health (MCH) clinics in Kuala Lumpur and Putrajaya. Participants were explained about the study and an informed consent was signed. A set of questionnaires in an electronic form was then administered to the participants. The questionnaire consists of two sections: (1) Sociodemographic details such as age, race, educational level, household income level, gestation week, and medical conditions, and (2) CPQ-P, which is the studied questionnaire provided in both English and Malay language. After two weeks of initial completion, the participants were contacted to complete the same CPQ-P again for test-retest analysis. The responses collected were checked for completeness and the participants were contacted if there were any missing answers. The validation of the CPQ-P consists of content validity, construct validity, and reliability (internal consistency and test-retest reliability) analyses.

### Modification of the questionnaire

The original CPQ was developed by Veronda and colleagues after conducting a literature search of existing measures of chrononutrition profile [22]. It consists of 19 items addressing the 6 main behaviours of chrononutrition, namely breakfast skipping, largest meal, evening eating, evening latency, night eating, and eating window. The CPQ has incorporated elements of sleep-wake rhythm synchronization between workdays and freedays from the Munich ChronoType Questionnaire (MCTQ) and preferences items from the Composite Scale of Morningness (CSM) [22]. To modify the original CPQ so that it can be relevant among the pregnant women population, we have conducted a literature search of factors affecting sleep-wake rhythms during pregnancy. The National Sleep Foundation’s Women and Sleep Survey reported that 78% of pregnant women experience disturbed sleep [23]. This condition may be due to pregnancy-related physical symptoms such as nausea, backpain, insomnia, and leg cramps, and will worsen as the women enters the third trimester of pregnancy [24, 25]. Therefore, a section asking on whether pregnancy-related symptoms would affect wake time, sleep time, and first meal time was added into the questionnaire. Additionally, daytime sleepiness was reported with a high prevalence of up to 84% during pregnancy and this may affect sleep timing [26, 27]. Pregnant women should have sufficient rest and at the same time do not exceed the recommended duration to avoid nocturnal sleep disturbance [28]. As sleep quality affects mother and infant outcome such as increased risk of caesarean section and preterm birth, we should consider sleep as an important element and explore the underlying factors causing poor sleep quality, including daytime napping [14]. Excessive daytime sleepiness has resulted in napping habits among pregnant women, therefore, the CPQ-P included questions about daytime napping to assess the frequency, timing, and duration of naps. Lastly, the Recommended Nutrient Intakes of Malaysia states that pregnant women should increase energy intake by 80–470 kcal per day across trimesters to accommodate for the growth of fetus and maternal tissues as well as maintaining a healthy gestational weight [29]. Pregnant women were recommended to consume small frequent meals while making healthier choices in selecting nutrient-dense foods [30]. Kebbe et al. (2021) reported that pregnant women tend to snack more frequently, especially closer to bedtime compared to before pregnancy [31]. Night time snacking has been found to negatively affect blood glucose regulation, sleep quality, and birth outcomes [32, 33]. Therefore, items addressing snacking frequency and timing were added into the CPQ-P.

### Content validity

Content validity is the extent to which the instrument fully represents the measured construct [34]. A content validation form was prepared, and four experts were invited to evaluate the items in the questionnaire in terms of their relevance, clarity, simplicity, and ambiguity. The expert panel consists of two nutritionists proficient in maternal and infant nutrition, and two nutritionist specializing in chrononutrition research. An online video meeting was conducted to present the questionnaire and collect feedback from the experts. The experts were required to fill in the content validation form which consists of a four-point Likert scale for the four categories of evaluation stated above for each item. The content validity index (CVI) was calculated from the responses collected, which ranges from one (not relevant) to four (very relevant). A score of 1 and 2 indicates the item was identified as not relevant, while score of 3 and 4 were classified as relevant items and suitable to be included in the questionnaire. A score of ‘1’ was assigned for items that were classified as relevant and achieved universal agreement (UA). Comments for each item was also collected from the experts and the questionnaire was revised accordingly.

### Construct validity

To determine the construct structure, present in the CPQ-P, an exploratory factor analysis (EFA) with varimax rotation was conducted for 29 items included in the questionnaire. Each item was labelled accordingly to identify its factor as shown in Table 1. The variables which indicate time (A1, A2, A3, A4, A5, A6, B1, B2, B3, C1, C2, C3, C4, C5, G1, and G2) which original format was hh:mm was multiplied by 24 h to show the numerical form of timing for the analysis. Variables A7, D3, D4, E1, E2, E3 were reported as frequency of days per week, while D1 and D2 were reported as frequency per day. Largest meal was reported as binary variables with each response separated into three categories: F1, F2, and F3.

**Table 1:** Items included in the questionnaire labelled according to their constructs

Label	Items
A1	Preferred wake time
A2	Weekday wake time
A3	Weekend wake time
A4	Preferred first eating event time
A5	Weekday first eating event time
A6	Weekend first eating event time
A7	Breakfast skipping
B1	Preferred fall asleep time
B2	Weekday bedtime
B3	Weekend bedtime
C1	Weekday dinner time
C2	Weekend dinner time
C3	Preferred last eating event time
C4	Weekday last eating event time
C5	Weekend last eating event time
D1	Weekday snacking frequency
D2	Weekend snacking frequency
D3	Snacking after last meal
D4	Night eating
E1	Pregnancy effect on wake time
E2	Pregnancy effect on sleep time
E3	Pregnancy effect on meal time
F1	Lunch as largest meal
F2	Breakfast as largest meal
F3	Dinner as largest meal
G1^a^	Weekday lunch time
G2 ^a^	Weekend lunch time
G3 ^a^	Daytime napping frequency
G4 ^a^	Daytime napping duration

^a^Items were excluded from questionnaire due to insufficient factor loading

Then, a confirmatory factor analysis (CFA) using maximum likelihood method was conducted to determine the construct validity. A path diagram was constructed using Amos Version 24 to test the goodness of fit of the hypothesized model, which specified correlated factors, and factor loading of item with highest discrepancy in each constructs set to 1.

### Reliability testing

An instrument is considered reliable if it produces a consistent result across time [35]. For this study, reliability was tested by determining the internal consistency and test-retest reliability. The Cronbach’s alpha indicates the internal consistency which is the extent of which the items in the questionnaire are inter-correlated, or consistent in measuring the same construct [36]. Test-retest reliability was conducted to determine the stability of responses from participants over a period of time across a repeated administration of the same questionnaire [36]. Test-retest reliability was evaluated using intraclass correlation coefficient (ICC).

### Statistical analysis

Sociodemographic data were presented as frequency (percentage) for categorical data and mean ± standard deviation for continuous data. For content validity, the S-CVI/UA was calculated by getting the average of universal agreement scores across all items. Shi et al. recommends that the cut off value for S-CVI/UA should be of 0.9 or higher for excellent content validity [37].

Exploratory Factor Analysis (EFA) was deployed to determine the structure presence across the items in CPQ-P. The Bartlett’s Test of Sphericity and Kaiser-Meyer-Olkin Measure of Sampling Adequacy were used to confirm the data was suitable for factor analysis. EFA was performed using varimax rotation, using maximum likelihood extraction and eigenvalues > 1. Items that loaded on different factors were grouped into theoretical relevant factors, while items with factor loading less than 0.298 were deleted from the questionnaire [38]. After determining the factor structure, confirmatory factor analysis using the maximum likelihood method was conducted to confirm the fit of variable into the model structure [39]. Model fit was determined using the following indices: normed chi-square (χ^2^/df); goodness of fit index (GFI); comparative fit index (CFI); and root mean-square error of approximation (RMSEA). A value of below 0.3 indicates a good fitting model, while values more than 0.9 for CFI and GFI is ideal to represent good fitting of the model [40]. RMSEA values should be more than 0.8 to be acceptable for the model fit [41]. Factor loading was also assessed for the model, which items with factor loading less than 0.298 will be considered for removal [38].

For reliability testing, internal consistency was determined using Cronbach’s alpha while test-retest reliability was examined using intraclass correlation coefficient. Cronbach’s alpha value above 0.60 is considered to have moderate level of reliability [42]. Intraclass correlation coefficient values less than 0.40 indicates poor reliability, while values between 0.40 and 0.59 indicates fair reliability, values between 0.60 and 0.74 indicates good reliability, and values above 0.75 were considered to have excellent reliability [43].

## Results

In total, 399 pregnant women participated in the validation of the CPQ-P. Most of them were Malay (85.5%), had tertiary level education (79.5%), and from moderate household income families (58.5%) as shown in Table 2. The mean pre-pregnancy body mass index (BMI) was 25.16 ± 6.01 kg/m^2^, with 48% of the pregnant women having normal pre-pregnancy BMI.

**Table 2:** Sociodemographic details of participants (*N* = 399)

Variable	N(%)
Age (years)^a^	31.68 ± 4.60
Gestational weeks^a^	25.49 ± 8.40
**Race**	
Malay Chinese Indian Others	342 (85.5) 32 (8.0) 15 (3.8) 11 (2.8)
**Educational level**	
None Primary Secondary Tertiary	5 (1.3) 7 (1.8) 70 (17.5) 318 (79.5)
**Household income**	
Low Moderate High	61 (15.3) 234 (58.5) 105 (26.3)
Pre-pregnancy BMI^a^ Underweight Normal Overweight Obese	25.16 ± 6.01 29 (9.0) 155 (48.0) 81 (25.1) 58 (18.0)

^a^Presented as mean ± SD

### Content validity

Table 3 shows the summary of content validity and modification of the questionnaire based on comments from the expert panel. From the original questionnaire, there is a lack of examples for the term ‘eating event’, hence a cultural relevant example was added at the end of the sentence which is “(e.g. Nasi lemak, biscuit/cookies, a cup of Milo)”. The expert panels also find it hard to identify “What time do you fall asleep?” since it may be unintentional. Hence, the panel has agreed to change the question to “What time do you go to bed?”. Other than that, the panel has found it confusing to give a standard answer for questions with regards to timing. Hence, an additional instruction “Please indicate A.M./P.M. as part of your response.” was added for items that required time as an answer. The comments from the expert panels had been addressed and modified accordingly in the CPQ-P questionnaire, hence an overall CVI of 1 was deemed appropriate for content validity (Table 4).

**Table 3:** Summary of comments and modifications for content and face validation based on comments from experts and target population (*n* = 4)

No.	Comments	Before modification	After modification
1	To give examples to define eating event	The original definition was stated as “The term ‘eating event’ refers to any time you eat something that contains calories. For example, this could be a meal, a snack, or a drink.”	Examples of eating events was added after the definition “The term ‘eating event’ refers to any time you eat something that contains calories. For example, this could be a meal, a snack, or a drink. (e.g. Nasi lemak, biscuit/cookies, a cup of Milo)”
2	Usage of terms to define sleep	The original item was “What time do you fall asleep?”	It has been modified to “What time do you go to bed?”
3	To simplify the statement for better understanding	The original item was stated “How often do you wake up in the night to eat?”	It has been simplified to “How often do you wake up at night to eat?”
4	Correcting the usage of grammar	The original item was “How often do you take naps in the day?”	It has been modified to “How often do you take naps during the day?”
5	To indicate answer must be in hours and minutes. HH:MM	No instructions for answering format of time was given.	The statement “Please indicate A.M./P.M. as part of your response.” was added for items that required time as an answer.

**Table 4:** Content validity index for CPQ-P by expert panel (*n* = 4)

Item descriptions	Total number of items	UA	S-CVI/UA	Interpretation
Relevance	36	35	0.97	Appropriate
Clarity	36	31	0.86	Appropriate
Simplicity	36	33	0.92	Appropriate
Ambiguity	36	32	0.89	Appropriate
		Average CVI	0.91	
		Overall CVI	1	Appropriate

S-CVI/UA > 0.9 = excellent content validity

### Construct validity

The summary of construct validity of 25 items in the CPQ-P using exploratory factor analysis with varimax rotation is shown in Table 5. The Kaiser-Meyer-Olkin Measure of Sampling Adequacy was 0.713 and the Bartlett’s Test of Sphericity was significant (*p* < 0.001), indicating that the data was suitable for EFA with sufficient samples and variables are correlated with each other. CPQ-P presented six factors and explained overall variances by 59.51% [44]. The six factors were labelled as Morning chrono-habits, Sleeping habits, Evening eating, Temporal eating habits, Pregnancy symptoms, and Largest meal. In the rotated factor matrix, the percentage of variances ranged from 6.59 to 19.21%. Each factor explained as shown: Factor 1 (Morning chrono-habits), 15.71%, Factor 2 (Sleeping habits), 10.93%, Factor 3 (Evening eating), 8.84%, Factor 4 (Temporal eating habits), 8.26%, Factor 5 (Pregnancy symptoms), 8.02%, and Factor 6 (Largest meal), 7.76%.

**Table 5:** Summary of the construct validity using EFA with varimax rotation (*n* = 358)

Items		Loading on 6 factors					
		1	2	3	4	5	6
		Morning chrono-habits	Sleeping habits	Evening eating	Temporal eating habits	Pregnancy symptoms	Largest meal
A1	Preferred wake time	0.812					
A2	Weekday wake time	0.788					
A3	Weekend wake time	0.773					
A4	Preferred first eating event time	0.759					
A5	Weekday first eating event time	0.758					
A6	Weekend first eating event time	0.690					
A7	Breakfast skipping	0.359					
B1	Preferred fall asleep time		0.899				
B2	Weekday bedtime		0.894				
B3	Weekend bedtime		0.850				
C1	Weekday dinner time			0.850			
C2	Weekend dinner time			0.781			
C3	Preferred last eating event time			0.584			
C4	Weekday last eating event time			0.577			
C5	Weekend last eating event time			0.392			
D1	Weekday snacking frequency				0.873		
D2	Weekend snacking frequency				0.847		
D3	Snacking after last meal				0.533		
D4	Night eating				0.453		
E1	Pregnancy effect on wake up time					0.879	
E2	Pregnancy effect on sleep time					0.852	
E3	Pregnancy effect on meal time					0.626	
F1	Lunch as largest meal						0.966
F2	Breakfast as largest meal						0.838
F3	Dinner as largest meal						0.382

Items with factor loading ≥ 0.298 are shown (Field 2013). The six factors presented the structure CPQ-P and explained 59.51% of the total variances. Factor 1 explained 15.71% of the total variances, factor 2 explained 10.93% of the total variances, factor 3 explained 8.84% of the total variances, factor 4 explained 8.26% of the total variances, factor 5 explained 8.02% of the total variances and factor 6 explained 7.76% of the total variances

Lunch time variables for weekdays and weekends did not have enough factor loading, with 0.253 and 0.167 respectively. Additionally, variables related to daytime napping such as ‘Daytime napping frequency’ and ‘Daytime napping duration’ had a factor loading of 0.188 and 0.142 respectively. This indicated that the lunch time variables and daytime napping variables were not meaningful enough did not strongly represent the any of the extracted factors, thus, it was removed from the CPQ-P.

**Fig. 1:**
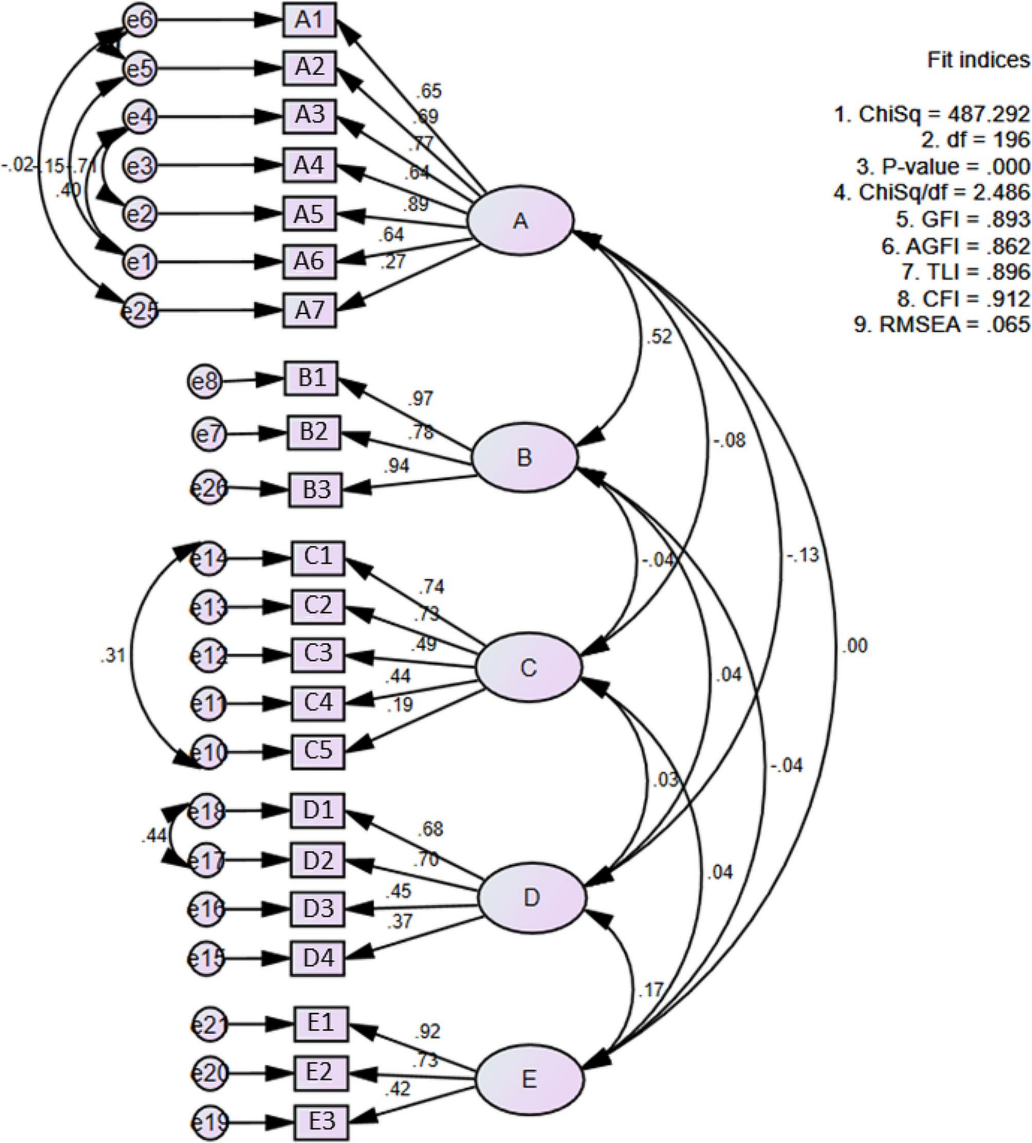
Factor model of the Chrononutrition Profile Questionnaire-Pregnancy (CPQ-P) with standardized estimates

Next, the hypothesized model was inserted for model fit testing using confirmatory factor analysis and showed poor fit (χ^2^/df = 4.408, CFI = 0.788, RMSEA = 0.098), and the factor loadings for the factor F, ‘Largest meal’ was weak (< 0.298). Hence, a second model which includes the correlations between errors was constructed for the 7 pairs with highest modification indices (e6-e5, e2-e4, e1-e4, e1-e5, e25-e6, e10-e14, e17-e18). Factor F, ‘Largest meal’, was also removed from the model. Figure 1 shows the factor model of the Chrononutrition Profile Questionnaire-Pregnancy (CPQ-P). In this model, the normed chi-square (χ^2^/df) was 2.486, indicating a good fit in the model as it was below 3. The GFI was 0.893, which was close to 0.9 and acceptable for research purposes [45]. The CFI of the model was 0.912, which is above cut-off of 0.9, indicating good fit of the model. Lastly, the RMSEA was 0.065, indicating optimal goodness of fit to the model. Notably, all variables had sufficient factor loadings except for A7- Breakfast skipping with factor loading close to cut-off of 0.289 (0.27) and C5- Weekend last eating event time with weak factor loading of 0.19.

### Reliability testing

A summary of internal consistency and test-retest reliability was depicted in Table 6. Cronbach’s alpha reliability coefficient (CAC) was examined to test the internal consistency of CPQ-P. The current CPQ-P had shown CAC of 0.612 to 0.923 among the 6 constructs of the questionnaire, indicating moderate to excellent internal consistency [42].

**Table 6:** Summary of test-retest reliability and internal consistency for CPQ-P after duration of 14 days

Item	Mean ± SD or Median ± IQR		Test-retest reliability		Internal consistency
	First test (*n* = 30)	Second test (*n* = 30)	ICC (*n* = 30)	95% CI	
**A. Morning chrono-habits**					0.824
A1. Preferred wake time	7:20 ± 1:36	7:06 ± 1:36	0.969*	0.93–0.99	
A2. Weekday wake time	6:39 ± 1:08	6:36 ± 1:06	0.975*	0.95–0.99	
A3. Weekend wake time	7:35 ± 1:43	7:43 ± 1:53	0.947*	0.89–0.98	
A4. Preferred first eating event time	8:54 ± 1:41	8:43 ± 1:43	0.984*	0.97–0.93	
A5. Weekday first eating event time	8:15 ± 1:54	8:23 ± 1:05	0.620*	0.20–0.82	
A6. Weekend first eating event time	9:20 ± 3:05	9:18 ± 2:53	0.948*	0.89–0.98	
A7. Breakfast skipping	0.80 ± 1.73	0.96 ± 1.88	0.661*	0.29–0.84	
**B. Sleeping habits**					0.923
B1. Preferred fall asleep time	18:00 ± 9:10	18:24 ± 8.33	0.845*	0.67–0.93	
B2. Weekday bedtime	15:43 ± 10:26	16:13 ± 10:04	0.889*	0.77–0.95	
B3. Weekend bedtime	12:52 ± 11:17	14:45 ± 10:48	0.818*	0.62–0.91	
**C. Evening eating**					0.612
C1. Weekday dinner time	19:32 ± 3:45	19:43 ± 2:12	0.917*	0.83–0.96	
C2. Weekend dinner time	19:59 ± 3:53	20:13 ± 0:40	0.540*	0.03–0.78	
C3. Preferred last eating event time	20:55 ± 1:03	20:58 ± 1:05	0.919*	0.83–0.96	
C4. Weekday last eating event time	20:41 ± 4:00	21:23 ± 0:52	0.793*	0.61–0.89	
C5. Weekend last eating event time	19:53 ± 4:01	21:11 ± 0.57	0.552*	0.06–0.79	
**D. Temporal eating habits**					0.615
D1. Weekday snacking frequency	2.10 ± 0.84	2.40 ± 1.25	0.620*	0.20–0.82	
D2. Weekend snacking frequency	2.20 ± 1.06	1.97 ± 0.96	0.731*	0.43–0.87	
D3. Snacking after last meal	3.20 ± 2.19	3.00 ± 1.29	0.422*	-0.21–0.73	
D4. Night eating	0.90 ± 2.07	0.63 ± 1.43	0.790*	0.56–0.90	
**E. Pregnancy symptoms**					0.714
E1. Pregnancy effect on wake time	2.80 ± 2.52	2.83 ± 2.76	0.637*	0.13–0.83	
E2. Pregnancy effect on sleep time	3.67 ± 2.88	3.40 ± 2.87	0.892*	0.77–0.95	
E3. Pregnancy effect on meal time	2.10 ± 2.50	2.40 ± 2.44	0.492	-0.07–0.76	
**F. Largest meal**					0.69
F1. Lunch as largest meal	0.60 ± 0.50	0.10 ± 0.30	0.39	-0.28–0.71	
F2. Breakfast as largest meal	0.93 ± 0.25	0.87 ± 0.35	0.788*	0.55–0.90	
F3. Dinner as largest meal	0.83 ± 0.38	0.63 ± 0.49	0.725*	0.42–0.87	

*Significant at the *p* < 0.05 using Intraclass Correlation test

For test-retest reliability, a total of 30 participants completed the first CPQ-P and second CPQ-P within an average of 14.40 ± 1.89 days. The intraclass correlation coefficient (ICC) was calculated to examine the reliability of the administered CPQ-P over a period of 14 days. From morning chrono-habits, variables on wake time and first eating event time had excellent reliability (ICC = 0.947–0.984), except for weekday first eating event time and breakfast skipping with good reliability (ICC = 0.620 and 0.661). In terms of sleeping habits, the ICC of variables for sleep time and bedtime had excellent reliability (0.818–0.889). From the evening eating construct, variables for dinner time and last eating event time had excellent reliability with ICC ranging from 0.793 to 0.919, except for weekend dinner time and weekend last eating event time with fair reliability of ICC value of 0.540 and 0.552 respectively.

In temporal eating habits, variables had good to excellent reliability, with night eating (ICC = 0.790) of excellent reliability, and weekday snacking frequency (ICC = 0.620) and weekend snacking frequency (ICC = 0.731) with good reliability, while snacking after last meal was found to have fair reliability (ICC = 0.422). In terms of the pregnancy symptoms construct, the ICC values ranges from fair to excellent reliability. Pregnancy effect on sleep time had excellent reliability (ICC = 0.892), pregnancy effect on wake time had good reliability (ICC = 0.637), while pregnancy effect on first meal time had fair reliability (ICC = 0.492). From the Largest meal construct, breakfast and dinner variables were found to have good to excellent reliability (ICC = 0.288 and 0.725), while lunch variables had poor reliability with ICC value of 0.390.

## Discussion

Determining chrononutrition variables have always been unstandardized and requires a lot of resources to collect. The study revealed that the CPQ-P had successfully determined the various chrononutrition profile of pregnant women and had good validity with 22 items in a 5 factor structure. It has also shown moderate to excellent internal consistency and test-retest reliability for the scales. The current CPQ-P was also in line with established literature on chrononutrition during pregnancy. Habits such as snacking frequency has been added into the questionnaire which was relevant for pregnant women. Based on Recommended Nutrient Intakes for Malaysia, pregnant women are required to increase their energy intake to provide for the growth of fetus, placenta, and various maternal tissues, as well as changes for the maternal metabolism [29]. Hence, snacking was recommended for pregnant women to increase their energy intake, and at the same time improving their diet quality with regular food intake and healthy food choices [46].

From the construct validation analysis, daytime napping variables did not have sufficient factor loading to be considered significant in construct validity. From our current sample, we have observed majority of the pregnant women (70.5%) took naps on one day in a week only. This may be due to work and social commitments, in which the pregnant women could only rest more on their ‘free days’. Hence, future assessments should also consider including preferred nap time, and segregating the daytime nap habits into weekdays and weekends to specify daytime napping habits among pregnant women. Additionally, lunch time variables did not load in our 6 factor structure and hence excluded from the questionnaire. This may be due to the variability of lunch timing among pregnant women in Malaysia. Based on the findings from the Malaysian Adults Nutrition Survey (MANS), lunch was defined as the meal between breakfast and dinner eaten during mid-day between 12:30 h to 2:30 h [47]. However, our current sample had a lunchtime ranging from 11:00 h to 16:00 h, suggesting great variability for lunch time among pregnant women.

The original CPQ developed by Veronda et al. (2020) had determined 6 chrononutrition behaviours, namely breakfast skipping, largest meal, evening eating, night eating, and eating window [22]. It has shown good convergent validity with the Automated Self-Administered 24-Hour Dietary Assessment Tool (ASA24) in reporting chrononutrition behaviours with strong correlation (*r* = 0.28 to 0.44). However, largest meal was shown to have poor validity in the original CPQ (Kappa = 0.012), which we had consistent results from CFA which factor loadings were not presented in the factor structure. It was recommended to include two or more questions measuring a single latent factor to provide meaningful statistical information on shared variance [48]. Hence, more information about largest meal consumption in a week could be collected by asking questions such as ‘In a 7-day period, how often do you take breakfast as your largest meal’, to provide a more comprehensive information about this construct. Other than that, breakfast skipping was also found to have low factor loading from our CFA, despite obtaining a good fit in the model (CFI = 0.912). Breakfast eating may not be accurate in this study as ‘breakfast’ was not well defined in the questionnaire. The amount of food and time period should be stated clearly to capture accurate data of breakfast eating, such as ‘first meal of the day consumed within 2 to 3 hours of waking, and comprised of food from at least 1 food group’ [49]. From the test-retest reliability analysis of the original CPQ showed low correlation among free days variables, suggesting that chrononutrition habits during free days are more variable over time. This finding was also evident in our analysis as weekend last eating event time had a weak factor loading of 0.19 and fair level of reliability with ICC value 0.552. Despite the weak statistical fitness of the variables in the CFA, the authors mutually agree that largest meal, breakfast skipping, and weekend last eating event time should be retained in the CPQ-P as it is of substantive interest to address chrononutrition habits [50]. Considering the significance of caloric intake in the day with weight status and diet quality during pregnancy [51], these items contribute meaningful information for healthcare providers to assess the pregnant women chrononutrition habits and further conduct personalized interventions.

In terms of night snacking, there were various contributing factors reported, such as hunger, thirst, nausea, altered sleep patterns, and fetal movements, which were common experiences during pregnancy [52]. These pregnancy symptoms may not be consistent and pregnant women reported to have consumed food whenever they chose to instead of following a proper meal schedule. Hence, the erratic meal timings during late night had contributed to the fair consistency of night snacking in this questionnaire (ICC = 0.422).

The CPQ-P had addressed several factors related specifically to the pregnant population, such as snacking and pregnancy effects on chrononutrition behaviours. It has also been mutually agreed that the items were relevant to pregnant women and examples given in the questionnaire were culturally acceptable. This questionnaire could be applied in clinical settings to determine the chrononutrition profile of pregnant women. The data obtained could be used to identify problem areas of the pregnant women’s lifestyle habits such as late night snacking and breakfast skipping, as well as addressing pregnancy-related symptoms. Chrononutrition emerge as a modifiable risk factor by altering the timing of food intake, with past literature reporting the different effects of caloric intake on weight loss and metabolic responses at different times of the day [53]. Health issues can be addressed by determining the association of chrononutrition habits with gestational weight gain, biomedical markers, and nutritional status during pregnancy via data collected from this questionnaire and routine antenatal check-ups.

Despite that, several limitations of the study are worth noting. Firstly, the current study did not engage with participants to conduct face validity and criterion validity, thus, its accuracy to capture data may be affected. Next, this study was conducted among pregnant women in the urban area of Kuala Lumpur and Putrajaya only, hence it may not be generalized to other pregnant women in suburban and rural areas. Although daytime napping did not show significant associations in this questionnaire, it has established a basis for future research to deploy different techniques in determining daytime napping habits among pregnant women.

It was suggested that more research should be conducted to further examine the validity and reproducibility of the CPQ-P among pregnant women from countries with seasonal variation where light-dark cycles may vary and affect the lifestyle of pregnant women. Other than that, it would be ideal to assess the convergent validity of pregnancy-related symptoms with diet records with meal timing.

## Conclusion

In conclusion, the Chrononutrition Profile Questionnaire - Pregnancy (CPQ-P) has demonstrated preliminary feasibility for addressing chrononutrition habits during pregnancy. CPQ-P was established with positive comments from experts, produced good construct validity, moderate to good internal consistency, and moderate test-retest reliability. Future studies can be conducted to translate and test the reliability of the CPQ-P among pregnant women from different countries of various cultural background. CPQ-P can be applied in future research to explore novel findings related to the circadian rhythms and pregnancy outcomes.

### Electronic supplementary material

Below is the link to the electronic supplementary material.

Supplementary Material 1

## Data Availability

The participants of this study did not give written consent for their data to be shared publicly, so due to the sensitive nature of the research supporting data is not publicly available. Data are however available from the corresponding author upon reasonable request.
