# Supplementary material for: Development, validation, and reliability of the Chrononutrition Profile Questionnaire-Pregnancy (CPQ-P)
Source: BMC Pregnancy Childbirth. 2024 Mar 23;24:217. doi: 10.1186/s12884-024-06403-4 (PMC10960373; doi:10.1186/s12884-024-06403-4)
Supplement: Supplementary file 1 — Supplementary Material 1 [file 12884_2024_6403_MOESM1_ESM.docx]

Appendix I Modification of Chrononutrition Profile Questionnaire-Pregnancy

| Question | Modification | Remarks |
| --- | --- | --- |
| **Part A. If you were entirely free to plan your day,** | |  |
| What time would you prefer to wake up? Please indicate A.M. or P.M. as part of your response. | No changes. |  |
| How soon after waking up would you prefer to have your first eating event of the day? | No changes. |  |
| How soon before bed would you prefer to stop eating? | No changes. |  |
| What time would you prefer to fall asleep? Please indicate A.M. or P.M. as part of your response. | No changes. |  |
| **Part B. On average, in a typical week (a 7-day period),** | |  |
| How often do you eat breakfast? | No changes. |  |
| What is your largest meal of the day? | No changes. | Insufficient factor loading from CFA. Retained in questionnaire due to its significance on assessing chrononutrition habits of population. |
| How often do you eat a snack after your last meal of the day? | No changes. |  |
| How often do you wake up in the night to eat? | No changes. |  |
| At this stage of pregnancy, how often does pregnancy-related symptoms affect your wake up time? (e.g. morning sickness, insomnia, cravings, nausea, etc) | New item added. |  |
| At this stage of pregnancy, how often does pregnancy-related symptoms affect your night sleep time? | New item added. |  |
| At this stage of pregnancy, how often does pregnancy-related symptoms affect your first meal time? | New item added. |  |
| If you indicated a response in B5c, what is change in timing for your meals? | New item added. |  |
| How often do you take naps during the day? | New item added. | Excluded from CPQ-P due to insufficient factor loading. |
| What time do you take naps? Please indicate A.M./P.M. as part of your response. | New item added. | Excluded from CPQ-P due to insufficient factor loading. |
| What is the average duration for each of your naps? | New item added. | Excluded from CPQ-P due to insufficient factor loading. |
| **Part C. On average, on a typical workday or weekday,** | |  |
| What time do you wake up? Please indicate A.M./P.M. as part of your response. | No changes. |  |
| What time is your first eating event of the day? Please indicate A.M./P.M. as part of your response. | No changes. |  |
| What time do you eat lunch? Please indicate A.M./P.M. as part of your response. Select “I do not eat lunch” if you do not typically eat lunch. | New item added. | Excluded from CPQ-P due to insufficient factor loading. |
| How many times do you eat snacks in a day? (Snacks are food/ beverage intake containing calories reported outside of main meals) | New item added. |  |
| What time do you have your snacks? Please indicate A.M./P.M. as part of your response. | New item added. |  |
| What time do you eat dinner? Please indicate A.M./P.M. as part of your response. Select “I do not eat dinner” if you do not typically eat dinner. | New item added. |  |
| What time is your last eating event before bed? Please indicate A.M./P.M. as part of your response. | No changes. |  |
| What time do you fall asleep? Please indicate A.M./P.M. as part of your response. | Question rephrased:  What time do you go to bed? Please indicate A.M./P.M. as part of your response. |  |
| **Part D. On average, on a typical weekend day or free day (days that you’re not working),** | |  |
| What time do you wake up? Please indicate A.M./P.M. as part of your response. | No changes. |  |
| What time is your first eating event of the day? Please indicate A.M./P.M. as part of your response. | No changes. |  |
| What time do you eat lunch? Please indicate A.M./P.M. as part of your response. Select “I do not eat lunch” if you do not typically eat lunch. | New item added. | Excluded from CPQ-P due to insufficient factor loading. |
| How many times do you eat snacks in a day? (Snacks are food/ beverage intake containing calories reported outside of main meals) | New item added. |  |
| What time do you have your snacks? Please indicate A.M./P.M. as part of your response. | New item added. |  |
| What time do you eat dinner? Please indicate A.M./P.M. as part of your response. Select “I do not eat dinner” if you do not typically eat dinner. | New item added. |  |
| What time is your last eating event before bed? Please indicate A.M./P.M. as part of your response. | No changes. |  |
| What time do you fall asleep? Please indicate A.M./P.M. as part of your response. | Question rephrased:  What time do you go to bed? Please indicate A.M./P.M. as part of your response. |  |
